# Phosphate-dependent aggregation of [KL]_n_ peptides affects their membranolytic activity

**DOI:** 10.1038/s41598-020-69162-0

**Published:** 2020-07-23

**Authors:** Erik Strandberg, Fabian Schweigardt, Parvesh Wadhwani, Jochen Bürck, Johannes Reichert, Haroldo L. P. Cravo, Luisa Burger, Anne S. Ulrich

**Affiliations:** 1 Institute of Biological Interfaces (IBG-2), Karlsruhe Institute of Technology (KIT), POB 3640, 76021 Karlsruhe, Germany; 2 Institute of Organic Chemistry, KIT, Fritz-Haber-Weg 6, 76131 Karlsruhe, Germany; 3 Laboratório de Biofísica Molecular, Universidade de São Paulo, Ribeirão Preto, SP Brazil

**Keywords:** Peptides, Membrane biophysics, Membrane structure and assembly, Biochemistry, Biophysics

## Abstract

In this study, we investigate how the length of amphiphilic β-sheet forming peptides affects their interaction with membranes. Four polycationic model peptides with lengths from 6 to 18 amino acids were constructed from simple Lys-Leu repeats, giving [KL]_n=3,5,7,9_. We found that (1) they exhibit a pronounced antimicrobial activity with an intriguing length dependent maximum for [KL]_5_ with 10 amino acids; (2) their hemolytic effect, on the other hand, increases steadily with peptide length. CD analysis (3) and TEM (4) show that all peptides-except for the short [KL]_3_-aggregate into amyloid-like fibrils in the presence of phosphate ions, which in turn has a critical effect on the results in (1) and (2). In fact, (5) vesicle leakage reveals an intrinsic membrane-perturbing activity (at constant peptide mass) of [KL]_5_ > [KL]_9_ > [KL]_7_ in phosphate buffer, which changes to [KL]_5_ ≈ [KL]_7_ ≈ [KL]_9_ in PIPES. A specific interaction with phosphate ions thus explains the subtle balance between two counteracting effects: phosphate-induced unproductive pre-aggregation in solution *versus* monomeric membrane binding and vigorous lipid perturbation due to self-assembly of the bound peptides within the bilayer. This knowledge can now be used to control and optimize the peptides in further applications.

## Introduction

Membrane-active antimicrobial peptides (AMPs) are found in almost all types of organisms, from bacteria to humans, as a host defense system against microorganisms^1,2^. Over 12,000 AMPs are known^3^. These peptides can be classified according to origin, activity and structure^1,4–6^. Cationic amphipathic α-helices are the most common, but another important class consists of cationic amphipathic β-sheet forming AMPs^7–9^.

Membrane activity is usually characterized in vivo using antimicrobial and hemolysis assays, and using biophysical studies in vitro, including vesicle leakage assays, as well as structural methods like circular dichroism and solid-state NMR. From these data, the mechanism of action can be elucidated, and it has been suggested that many amphipathic α-helices operate by forming pores in membranes. We have recently studied the length dependent membrane activity of α-helical AMPs using a series of model peptides with 14–28 amino acids, based on the underlying repeat unit [KIAGKIA]. These peptides were found to be active only when they are long enough to span a membrane, which enables them to flip upright within the lipid bilayer and form a transmembrane toroidal pore^10–13^.

Besides the α-helical [KIAGKIA]-based peptides, we have also focused on the sequence [KIGAKI]_3_, which has a similar composition but exhibits an underlying β-stranded amphiphilic pattern. The membrane-bound conformation of this AMP with 18 residues was characterized using solid-state NMR^14,15^. At low concentrations it binds to the membrane as a flexible monomer (with an intrinsically disordered conformation within the 2D plane of the lipid bilayer), but beyond a threshold concentration the flexible strands were found to self-assemble into immobilized β-sheets^14^. By including a single D-amino acid in the sequence, the tendency to form β-sheets could be reduced, which also lowered the hemolytic and fusogenic activity of the peptide, but not its antimicrobial effect^14,16^.

The effect of peptide length on the activity of amphiphilic β-stranded AMPs has already been described in several reports^9,17^. When the length dependence of membrane binding and folding into β-sheets of KIGAKI peptides was studied^18^, it was found that both binding and folding increases strongly with peptide length up to 18 amino acids. Here, we investigate a series of β-sheet forming AMPs with a very simple repetitive sequence [KL]_n_-NH_2_, using four peptides with 6, 10, 14 or 18 amino acids (n = 3, 5, 7, 9). We call them collectively KL peptides and individual peptides are given names according to the number of amino acids, for example KL10 for [KL]_5_-NH_2_ (see Table 1).

**Table 1:** Synthesized peptides used in this study.

Peptide	Sequence	Length (amino acids)	Net charge	Molecular weight (g/mol)
KL6	KLKLKL-NH_2_	6	+ 4	741.0
KL10	KLKLKLKLKL-NH_2_	10	+ 6	1,223.7
KL14	KLKLKLKLKLKLKL-NH_2_	14	+ 8	1706.3
KL18	KLKLKLKLKLKLKLKLKL-NH_2_	18	+ 10	2,189.0

It has long been known that KL peptides can form β-sheets in solution^19,20^, and it has been shown that the amount of β-structures increases with peptide concentration, with salt concentration^19,21^, and over time^22^. The goal of the present study was to obtain systematic insights into the repertoire of peptide structures that may be encountered under different conditions, in order to eventually find the functionally active state and describe the peptide-lipid interactions at the very moment of membrane destabilization. Namely, in the mid-term we would like to find out whether or not the β-stranded peptides are able to form transmembrane β-barrel pores, in analogy to the toroidal wormholes formed from α-helical AMPs. For such structural investigations, a thorough understanding of the relationship between membrane activity and β-sheet assembly is essential, and it is also necessary to establish the length dependence of the biological activity and the tendency to aggregate. Just like in our earlier study of α-helical KIAGKIA-based peptides^11–13^, here we use biological assays to determine the membranolytic effect of the β-stranded KL peptides on bacteria and erythrocytes, supplemented by vesicle leakage assays to check for perturbation of synthetic lipid vesicles, besides circular dichroism spectroscopy (CD) to characterize the peptide secondary structure. In addition, we used time-resolved CD to study the speed of β-sheet formation, and transmission electron microscopy to visualize fibril formation.

## Materials and methods

### Materials

Peptide synthesis reagents and Fmoc-protected amino acids were purchased from Iris Biotech (Marktredwitz, Germany) or Merck Biosciences (Darmstadt, Germany). Chemicals for peptide synthesis were obtained from Merck (Darmstadt, Germany) or Biosolve (Valkenswaard, Netherlands), and solvents for HPLC purification were purchased from Fischer Scientific (Geel, Belgium). 4-Chloro-7-nitrobenzofurazan (NBD-Cl) was purchased from VWR (Bruchsal, Germany). The fluorescent probes 8-amino-naphtalene-1,3,6-trisulfonic acid sodium salt (ANTS) and p-xylene-bis-pyridinium bromide (DPX) were obtained from Invitrogen-Molecular Probes (Karlsruhe, Germany). Fluorescently labelled 1,2-dioleoyl-*sn*-glycero-3-phosphoethanolamine-N-(lissamine rhodamine B sulfonyl) (Rhod-PE) was obtained from Avanti Polar Lipids (Alabaster, AL, USA). The lipids 1-palmitoyl-2-oleoyl-*sn*-glycero-3-phosphatidylcholine (POPC), 1-palmitoyl-2-oleoyl-*sn*-glycero-3-phosphatidylglycerol (POPG), 1,2-dimyristoyl-*sn*-glycero-3-phosphatidylcholine (DMPC), and 1,2-dimyristoyl-*sn*-glycero-3-phosphatidylglycerol (DMPG) were purchased from NOF (Grobbendonk, Belgium).

### Peptide synthesis

[KL]_n_–NH_2_ peptides were synthesized on an automated Syro II multiple peptide synthesizer (MultiSynTech, Witten, Germany) using standard Fmoc solid phase peptide synthesis protocols^23,24^. In addition to the normal unlabeled peptides, peptides were also synthesized with the fluorophore NBD attached to their N-terminus, in order to monitor vesicle binding using fluorescence spectroscopy. The crude peptides were purified on an HPLC system from JASCO (Groß-Umstadt, Germany) using a preparative Vydac C18 column with a water/acetonitrile gradient supplemented with 5 mM HCl. The purified peptides were characterized by analytical LC (Agilent, Waldbronn, Germany) coupled to an ESI mass spectrometer (µTOF Bruker, Bremen, Germany), and were found to be over 95% pure.

### Circular dichroism spectroscopy (CD)

#### Vesicle sample preparation

CD samples were prepared by co-solubilizing DMPC and DMPG (4/1 mol/mol) or POPC and POPG (1/1 mol/mol) in chloroform/methanol 1/1 (v/v). The organic solvent was evaporated with a gentle stream of nitrogen, and samples were then kept under vacuum for 3 h. The dried lipid film was dispersed in milliQ water and homogenized by 10 freeze–thaw cycles and vortexing. Small unilamellar vesicles (SUVs) for CD samples were generated by sonication for 16 min in a high-power ultrasonic bath with a beaker-shaped sonotrode (UTR 200, Hielscher, Germany).

#### Measurements

CD spectra were recorded on a J-815 spectropolarimeter (JASCO, Groß-Umstadt, Germany) between 260 and 185 nm at 0.1-nm intervals, using 1-mm quartz-glass cells (Suprasil; Hellma, Müllheim, Germany) as reported previously^14^. The peptides were measured at 25 °C in milliQ water and in 10 mM sodium phosphate buffer (PB, pH 7.0), and at 30 °C in the presence of lipid vesicles composed of DMPC/DMPG (4/1 mol/mol) or POPC/POPG (1/1 mol/mol). The typical peptide concentration of the final samples in milliQ water, phosphate buffer, and in the vesicle samples was 0.1 mg/mL (40–170 µM depending on the peptide molecular weight). The peptide-to-lipid (P/L) molar ratio was 1/20 for the KL14 peptide, and was adapted for the other peptide-lipid mixtures to get the same constant mass ratio for peptides and lipids. From each sample spectrum an averaged baseline was subtracted of the pure solvent or lipid matrix, respectively. Finally, the spectra were converted to mean residue ellipticities by using the weighed-in amount of peptide and the volume of the sample for calculating peptide concentration. A concentration measurement based on UV/VIS absorption spectroscopy of aromatic residues was not possible, because KL peptides do not contain any aromatic residues.

#### Deconvolution of CD data

**Figure 1:**
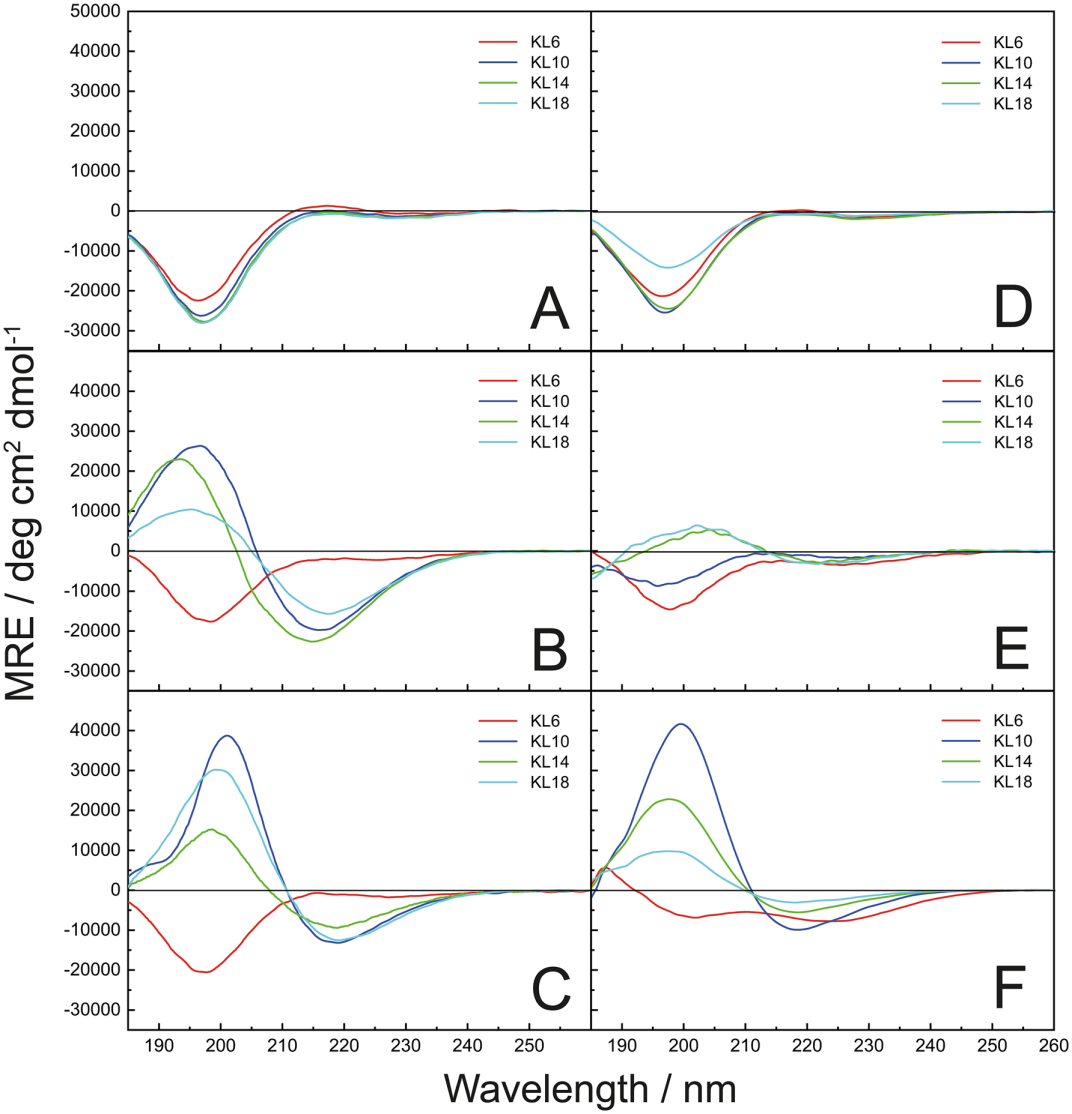
Circular dichroism spectra of KL peptides (compared at constant peptide mass). (**A**) In MilliQ water, pH ≈ 6. (**B**) In water, pH ≈ 10. (**C**) In 10 mM phosphate buffer, pH = 7.0. (**D**) In the presence of DMPC vesicles in water, pH ≈ 6. (**E**) In the presence of DMPC/DMPG (4/1) vesicles in water, pH ≈ 6. (**F**) In the presence of POPC/POPG (1/1) vesicles in water, pH ≈ 6.

A quantitative deconvolution of CD data to determine the relative contents of different secondary structure elements was performed exemplarily for the KL10 spectra in H_2_O, pH ≈ 10 and in 10 mM PB, pH = 7, where clear sample solutions without any spectral artifacts due to light scattering caused by aggregated particles had been observed (this is also valid for all the other KL peptide spectra shown in Fig. 1A–C). For secondary structure estimation the on-line version of the Beta Structure Selection (BeStSel) method was applied that takes into account the twist of β-structures and can reliably distinguish parallel and antiparallel β-sheets^25,26^. CD samples of KL peptides measured in lipid SUVs environment were always quite turbid, i.e. one cannot exclude significant differential scattering and absorption flattening artifacts, which did not permit a quantitative secondary structure estimation, due to the large errors encountered.

#### Time-dependent measurements

Time-resolved measurements were performed to determine the speed of aggregation of KL peptides in solution, as a function of the concentration of phosphate ions and other salts, and as a function of peptide length. Measurements were performed at a fixed wavelength of 200 nm with a time resolution of 1 s. Before starting a time scan, peptides were measured in MilliQ water in a cuvette with 10 mm optical path length, equipped with a magnetic stirrer (300 rpm), to get a starting point value for non-aggregated peptides. Phosphate buffer or salt solution was added to the cuvette to adjust a specified concentration, and shortly afterwards the time course measurement of the CD signal was triggered. The first 5–10 s after addition could not be measured due to the delay time it took for complete mixing, but curves were fitted with this time delay taken into account, and the known starting point of peptides in MilliQ water, measured separately before, was taken as the zero time point. Data were re-calculated as folding percentage, setting the starting point to 0% and the final maximum signal as 100%. Data were then fitted according to the exponential function according to Eq. (1):

1 $${\text{A}}({\text{t}}) = 1 - \exp ( - {\text{t/}}\uptau )$$

Here, t is the elapsed time from the addition of phosphate or salt, A(t) is the time-dependent proportion of β-sheet signal (with A(0) = 0), and τ is the time constant of β-sheet assembly.

### Transmission electron microscopy (TEM)

TEM images were obtained using a Philips CM120 and a Philips Tecnai F20 FEG electron microscope. The peptide solutions without phosphate buffer were aged 24 h before TEM sample preparation. Peptide solutions with PB (5 mM, pH = 7.0) were not allowed to age but always prepared freshly. For imaging, a 5 µL aliquot of the 2 mM peptide stock solution was applied on a copper grid with carbon film. After 1 min of adsorption, the sample was negatively stained with a 2% uranyl acetate solution in water. Excess staining solution was removed with moistened filter paper.

### Nuclear magnetic resonance (NMR) spectroscopy

Solid-state ^31^P-NMR experiments were performed on a Bruker Avance 500 MHz spectrometer (Bruker Biospin, Karlsruhe, Germany) at 308 K. A Hahn echo sequence was used with a phase cycling^27^ using a 3.9 μs 90° pulse, a 30 µs echo time, a 1 s relaxation delay time, and 13 kHz proton decoupling with a SPINAL-64 sequence^28^. 256 scans were recorded and 20 Hz line broadening was applied to the spectra.

### MIC (minimum inhibitory concentration) assay

Antimicrobial activity was measured by a standard minimal inhibitory concentration (MIC) assay, carried out with Gram-positive *Bacillus subtilis* subsp. *spizizenii* (DSM 374) and *Staphylococcus xylosus* (DSM 20267) and with Gram-negative *Escherichia coli* (DSM 1116) and *Enterobacter helveticus* (DSM 18390), as previously reported^14,29^. Bacteria were grown in Müller-Hinton medium (MHM) at 37 °C overnight. Microtiter plates (96 wells of 100 μL) were filled with 50 µL MHM and peptide solution to obtain serial twofold dilution series of peptides. The two final columns of each plate remained without peptide, so that the penultimate data point served as the positive control (no peptide), and the final one as the negative control (not inoculated). 50 μL of bacterial suspension (OD = 0.2) was added to the wells (except for the final column of each plate) to give a final concentration of 10^6^ CFU/mL. The plates were incubated at 37 °C for 20 h, and cell viability was probed by addition of 20 µL 0.2 mg/mL resazurin and incubation at 37 °C for 2 h. The MIC value was determined visually as the lowest peptide concentration inhibiting bacterial growth.

Two versions of the MIC assay are examined here for comparison, using different ways to prepare the two-fold peptide dilutions. Two different stock solutions of MHM were employed in version A and B, respectively. The regular MHM contained 3 g/L beef extract, 17.5 g/L acid hydrolysate of casein and 1.5 g/L starch, whilst the double concentrated MHM (from here on called 2 × MHM) contained 6 g/L beef extract, 35 g/L acid hydrolysate of casein and 3 g/L starch. In version A, the standard protocol used previously in our group, 50 µL MHM was added to each well, with a double concentration (2 × MHM) in the first well. To the first well, 50 µL peptide solution in MilliQ water was added and mixed. 50 µL of this was transferred to the second well, and 50 µL from the second well to the third well and so on. Some 50 µL from the last well were discarded. After all plates were prepared, 50 µL MHM with bacteria was added to each well, giving a total filled volume of 100 µL. In version B, 50 µL of MilliQ water was first added to each well. To the first well 50 µL peptide solution in MilliQ water was added. After mixing, 50 µL of this was transferred to the second well, and so on. After all plates were prepared, 50 µL 2 × MHM with bacteria was added to each well, giving again a total volume of 100 µL. The final composition of each well was the same in the two versions, but in version A the peptides were in contact with phosphate in the medium for at least 1 h before the bacteria were added, whereas in version B the peptides made contact with the phosphate ions and the bacteria simultaneously.

### Hemolysis assay

Hemolytic activity was examined using a serial twofold dilution assay as previously described^13,30^. Citrate phosphate dextrose-stabilized blood bags with erythrocyte suspensions of healthy donors were obtained from the blood bank of the local municipal hospital (Städtisches Klinikum, Karlsruhe, Germany). The erythrocytes were washed with Tris-HCl buffer with pH 7.6, and incubated with peptide solutions at 37 °C for 30 min with gentle shaking. The tubes were centrifuged at 13,000 rpm for 10 min to pellet the cells, and the absorbance at 540 nm was recorded against a negative control (cells without peptide, accounting for autohemolysis). The percentage of lysis was then calculated relative to 100% lysis induced by 1% Triton X-100. The absorbance measurements were repeated three times, and the averaged values were used.

### Vesicle leakage assay

#### Preparation of large unilamellar vesicles

For the leakage experiments^31,32^, the buffer in which the vesicles were prepared contained the fluorophore ANTS (12.5 mM), the quencher DPX (45 mM), 50 mM NaCl, and either 10 mM phosphate (pH 7.0) or 10 mM PIPES buffer (pH 7.0). Liposomes were prepared by co-dissolving POPC/POPG (1/2 mol/mol) lipids in CHCl_3_/MeOH (3/1 v/v), together with 10^–2^ mol% Rhod-PE (used as a marker to quantify the loss of lipids during the extrusion and gel filtration steps required for vesicle preparation, see below). The lipid mixture was dried under N_2_(g) and left to dry under vacuum overnight. The obtained thin film was then re-suspended in the buffer which contained the fluorophore and the quencher by vigorous vortexing, followed by 10 freeze–thaw cycles^33^. Large unilamellar vesicles (LUV) were obtained by 41-fold extrusion (Avanti Mini Extruder; Avanti Polar Lipids, Alabaster, AL) of the liposomes through a Nuclepore polycarbonate membrane (pore size 100 nm, Whatman-GE Healthcare Europe, Freiburg, Germany) at room temperature. Unencapsulated dye was removed by gel filtration using spin columns filled with Sephacryl 100-h (Sigma-Aldrich, Taufkirchen, Germany), and equilibrated with an elution buffer (150 mM NaCl, buffer agent, pH 7.5) which balances the internal vesicle osmolarity. Different buffer agents were used for the external buffer, and the order of mixing of peptides and vesicles was also varied, as described in the Results section.

#### Fluorescence dequenching assay

Leakage of entrapped ANTS was monitored by fluorescence dequenching of ANTS^34^. Fluorescence measurements were performed in a thermostated cuvette with constant stirring at 30 °C in the same buffer as used for gel filtration on a FluoroMax2 spectrofluorimeter (HORIBA Jobin Yvon, Unterhaching, Germany), by setting the ANTS emission to 510 nm (5 nm slit) and its excitation to 355 nm (5 nm slit). Large unilamellar vesicles (100 µM final lipid concentration) were either added to the cuvette containing a constant weight of 30 µg peptide (corresponding to a peptide-to-lipid molar ratio (P/L) of 1/10 for KL14), or peptides were added to the cuvette containing the vesicles. For comparison, another series of experiments was carried out in a more conventional way, using a constant molar concentration of peptide, i.e. 10 µM (corresponding to a P/L of 1/10 for all peptides), which means increasing mass with increasing peptide length. The level of 0% leakage corresponded to the fluorescence of the solution immediately after mixing peptides and vesicles, while 100% leakage was the fluorescence value obtained upon addition of 0.25 vol% Triton X-100 after 10 min.

### Vesicle binding assay

The assay to study the binding of peptides to vesicles is described in the Supporting Information.

## Results

### Peptide synthesis

KL peptides with the repetitive sequence [Lys-Leu]_n_-NH_2_ with 6, 10, 14 and 18 amino acids (n = 3, 5, 7, 9) were used in the study, as listed in Table 1. All peptides were successfully synthesized and purified with a purity of > 95% according to HPLC. Since the main topic of the study is the length dependent activity of KL peptides, we will use peptides names like KL10, to emphasize that this peptide contains 10 amino acids.

### Circular dichroism

The secondary structure of the KL peptides was investigated with circular dichroism spectroscopy (CD), using always the same total amount (weight) of peptide for comparing the different lengths. In deionized water all peptides gave CD spectra with a minimum around 197 nm, indicating a random coil conformation (Fig. 1A). Since these aqueous solutions were somewhat acidic due to residual HCl from HPLC purification in the lyophilized peptides, the pH in this case was around 6. When a small aliquot of 0.1 M NaOH was added to the solution, resulting in an approximate pH ≈ 10, KL6 is still random coil, but the longer peptides all gave spectra with a maximum around 195 nm and a minimum around 215 nm, indicating β-sheeted structures (Fig. 1B). Also in 10 mM phosphate buffer at pH = 7.0, KL6 is random coil, but the other peptides gave spectra with a slightly red-shifted maximum around 200 nm and a minimum near 219 nm, indicating more pronounced β-sheet structures for KL10 to KL18 (Fig. 1C). The KL peptides are designed to form amphipathic β-sheets when bound to membranes, so CD spectra were also measured in the presence of small unilamellar lipid vesicles. In vesicles of zwitterionic DMPC in water at pH ≈ 6 (Fig. 1D), all KL peptides show random coil spectra, indicating that the peptides do not bind to these neutral membranes. In a slightly anionic lipid mixture of DMPC/DMPG (4/1) in water at pH ≈ 6 (Fig. 1E), the CD spectra are less intense due to strong turbidity and flocculation in the aqueous lipid suspension, leading to differential scattering and absorption flattening spectral artifacts. The spectra reveal a mixture of random coil and β-sheet signals, with more β-sheet for the longer peptides. In POPC/POPG (1/1) vesicles with a high proportion of anionic lipids, in water at pH ≈ 6, KL10, KL14, and KL18 show once again β-sheets (Fig. 1F), similar to spectra in PB (Fig. 1C), while KL6 gives a different spectrum that can be interpreted as a superposition of random coil plus β-sheet signals. In these latter samples some turbidity was observed.

KL6 thus seems to be too short to form β-sheets in aqueous solution, but the longer peptides do so in water at pH ≈ 10. In the presence of phosphate buffer, peptides are also forming β-sheets but the line shapes are somewhat different, with maxima and minima red-shifted by ~ 5 nm compared to the aqueous solutions. The CD spectra of KL10 in these two cases were deconvoluted using the BeStSel algorithm^25,26^ to get an estimate of the secondary structure elements present. In PB, 100% β-sheets were found (83% antiparallel and 17% parallel). In water at pH ≈ 10, there was only a 54% contribution from β-sheets (34% antiparallel and 20% parallel), while the rest was helical plus other conformations. This deconvolution indicates that KL10 in water at pH ≈ 10 only partly forms β-sheets, but in PB the β-sheet formation is essentially complete.

Binding to neutral DMPC membranes is very weak, so these spectra are similar to those in water at pH ≈ 6 without lipid vesicles, where peptides are unstructured. In contrast, the polycationic peptides bind avidly to POPC/POPG (1/1) vesicles, giving similar CD spectra as in PB, which reflect complete β-sheet formation.

**Figure 2:**
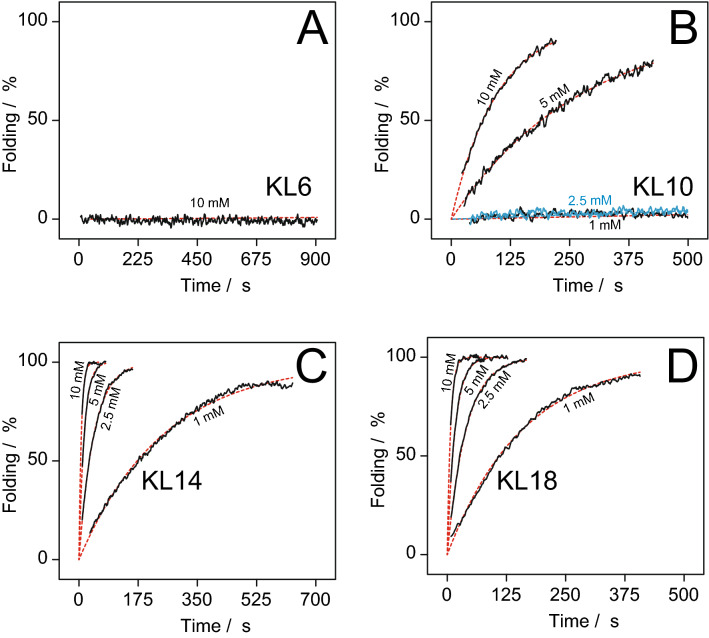
Percentage of peptide folded into β-sheets, calculated from the CD intensity at 200 nm, as a function of time and phosphate concentration in solution. Black curves are experimental data, dotted red lines are fits to Eq. (1). The peptide concentration was always 0.01 mg/mL, i.e. the same weight was used for the different peptide lengths. (**A**) KL6 shows no time dependent effect and maintains its random coil conformation throughout, even at the highest tested phosphate concentration of 10 mM. (**B**) KL10 does not change up to 2.5 mM phosphate, but at 5 and 10 mM phosphate is becomes assembled into β-sheets. In 10 mM phosphate, folding happens within 5 min. (**C**) KL14 folds already at a 1 mM concentration of phosphate, and at 10 mM folding is complete in less than a minute. (**D**) KL18 folds even faster than KL14.

The longer KL peptides are clearly able to form β-sheet aggregates in PB without lipid vesicles present. To investigate the rate of aggregation, time-resolved CD experiments were performed. The CD intensity at 200 nm was measured for up to 900 s after adding different concentrations of phosphate. Phosphate solution was added to a stirred peptide solution in water (pH ≈ 6), and the aggregation could be followed, as the signal at 200 nm is highly sensitive to a change from random coil (with a close-by minimum) to β-sheet (with a maximum in this region). An initial full spectrum was measured before addition of phosphate. The first few seconds, following the addition of peptides, could not be measured, but the time delay could be determined from a fit of the curve, which also gave the time constant of β-sheet formation. These time constants are listed in Table 2. KL6 did not show any change in CD signal over 600 s, even in 10 mM phosphate buffer (Fig. 2A). KL10 shows almost no folding up to 2.5 mM phosphate, but with 5 mM there is a distinct increase in signal with a time constant τ of 273 s, up to a maximum after around 600 s. At 10 mM, folding is faster with a time constant of 96 s. (Fig. 2B). After the maximum is reached, the signal starts to decrease again, indicating that peptide starts to fall out of solution whereupon the CD signal is lost for those precipitated molecules (data not shown). KL14 (Fig. 2C) starts to fold already at 1 mM phosphate, and in 10 mM phosphate buffer it has completely turned into β-sheets after just a few seconds, with a time constant of 7 s. KL18 shows a similar behavior as KL14, but with even faster kinetics (Fig. 2D). Clearly, aggregation into β-sheets is much faster for longer KL peptides and for higher concentrations of phosphate ions. Note that the absolute weight of the different KL peptides was the same in each sample.

**Table 2:** Time constants (in s) of random coil ↔ β-sheet transition for KL peptides in solution in the presence of phosphate.

Peptide	Phosphate concentration (mM)			
	1.0	2.5	5.0	10.0
KL6^a^	∞	∞	∞	∞
KL10	23,500	12,900	273	96
KL14	248	44	15.5	7.0
KL18	158	39.5	16.5	7.0

^a^For KL6 no folding was observed after 900 s; τ values could not be fitted properly but are essentially infinite.

**Figure 3:**
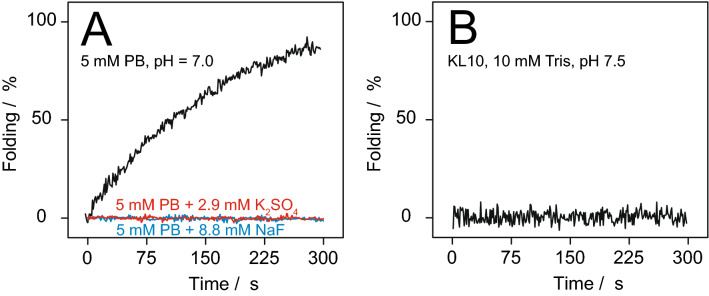
Percentage of peptide folded into β-sheets, calculated from the CD intensity at 200 nm, as a function of time for KL10 under different conditions. (**A**) In 5 mM PB there is fast aggregation, but when salt is added (either 8.8 mM NaF or 2.9 mM K_2_SO_4_) there is no aggregation. (**B**) In 10 mM Tris buffer there is no aggregation.

To investigate whether the dramatic influence of phosphate buffer was specific for this type of polyvalent anion, or rather a more general effect of salt concentration or pH, additional time series were measured. Instead of phosphate, KL10 was dissolved in water in the presence of NaF (with monovalent ions) or K_2_SO_4_ (with divalent SO_4_^2−^ ions). To get the same ionic strength as 5 mM phosphate at pH = 7, 8.8 mM NaF and 2.9 mM K_2_SO_4_ were used, respectively. In both cases, no aggregation was observed over 900 s. These salts have no buffering capacity, and the pH of these solutions was around 6. Therefore, the salts were also added to 10 mM PB at pH 7.0. As seen in Fig. 3A, the addition of salt actually blocked the effect of the phosphate ions, and no aggregation was seen. Using instead of phosphate buffer a Tris buffer with pH = 7.5, also no aggregation was observed (Fig. 3B). Changing the pH of the Tris buffer to 8.0, or adding NaF or K_2_SO_4_ to the Tris buffer, also gave no aggregation (data not shown). Thus, aggregation is dramatically enhanced specifically in the presence of phosphate ions, whereas the addition of other salts can block this effect, presumably by shielding off the underlying electrostatic interactions.

### TEM

**Figure 4:**
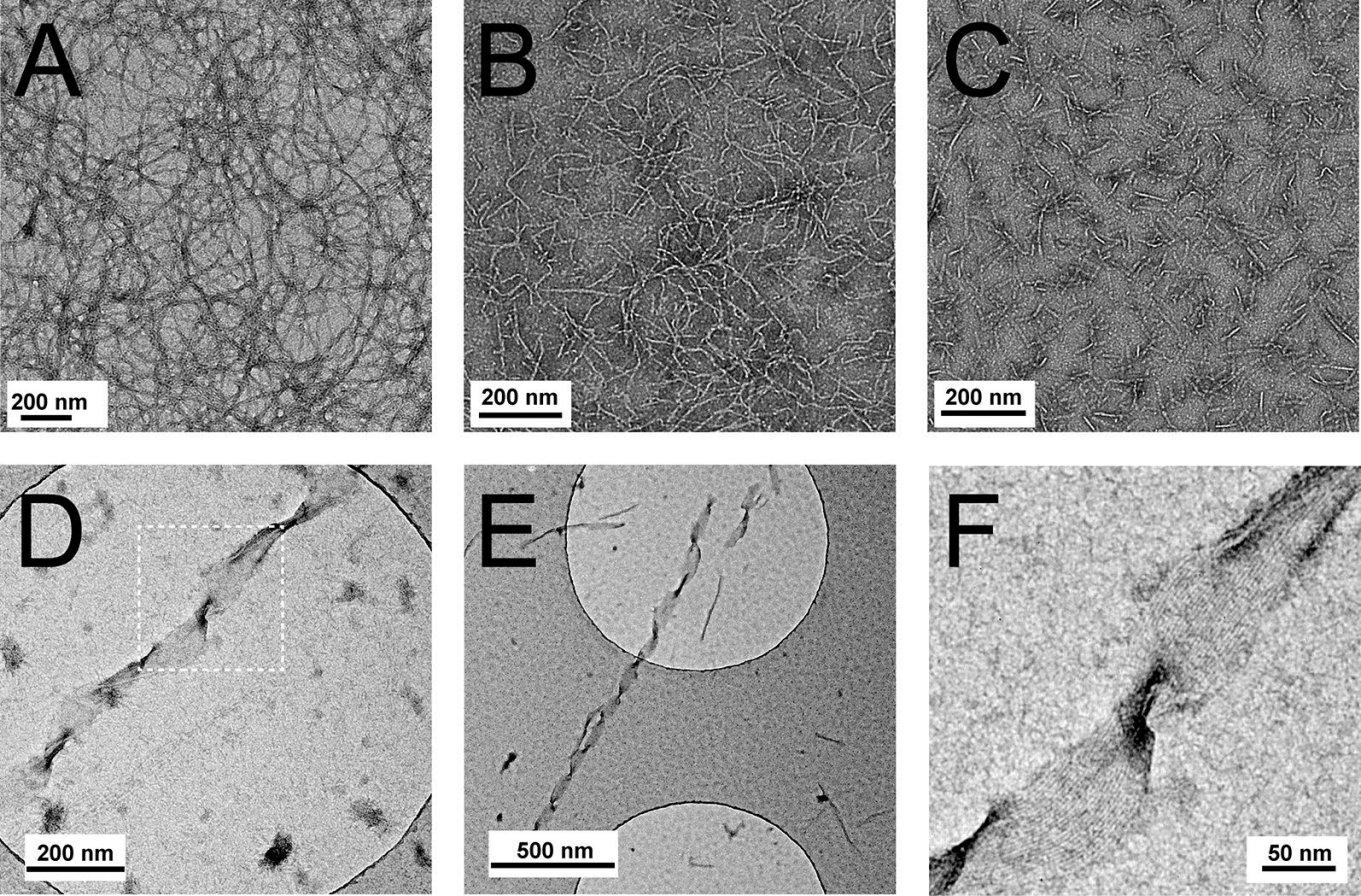
TEM images of aggregated KL peptides: (**A**) KL10, (**B**) KL14, (**C**) KL18, showing fibrils in water at pH ≈ 10 after aging 24 h. (**D**–**F**) TEM pictures of KL peptides from 2 mM peptide solutions in 5 mM PB, not aged. (**D**) KL14 in 5 mM PB, pH = 7.0. (**E**) KL18 in 5 mM PB pH = 7.0. (**F**) Enlargement of the twisted fibril in the marked area in (**D**), showing parallel lines with a regular spacing of about 3 nm.

TEM images were recorded of KL10, KL14 and KL18 peptides in water. KL6 did not show any aggregation in CD experiments and was therefore not included in the TEM study. Peptides solutions were prepared in MilliQ water at pH ≈ 10, and samples were aged for 24 h before being deposited on the grid where images were taken. As seen in Fig. 4A, KL10 forms distinct fibrils in water at pH ≈ 10, with a thickness estimated to be 20–30 nm. Also KL14 (Fig. 4B) and KL18 (Fig. 4C) form fibrils. When the KL10 sample was sonicated, broken fragments were observed (Fig. S1), showing that these thin fibrils are not very stable.

Another series of peptide samples was prepared in 10 mM phosphate buffer at pH = 7.0, and in this case samples were prepared freshly just before being deposited on the grid. Again, all three peptides formed fibrils, and these were found to be assembled further into twisted ribbons, as illustrated for KL14 and KL18 (Fig. 4D, E). An analysis of the high resolution image of KL14 (Fig. 4F) reveals that these ribbons consist of several parallel proto-fibrils with a spacing of around 30 Å. This is similar to the length of this peptide in an extended β-stranded conformation (14 amino acids × 3.5 Å ≈ 50 Å), possibly seen from an angle. These findings suggest that the aggregated KL peptides form amyloid-type cross-β-sheet fibrils.

### Antimicrobial activity

**Figure 5:**
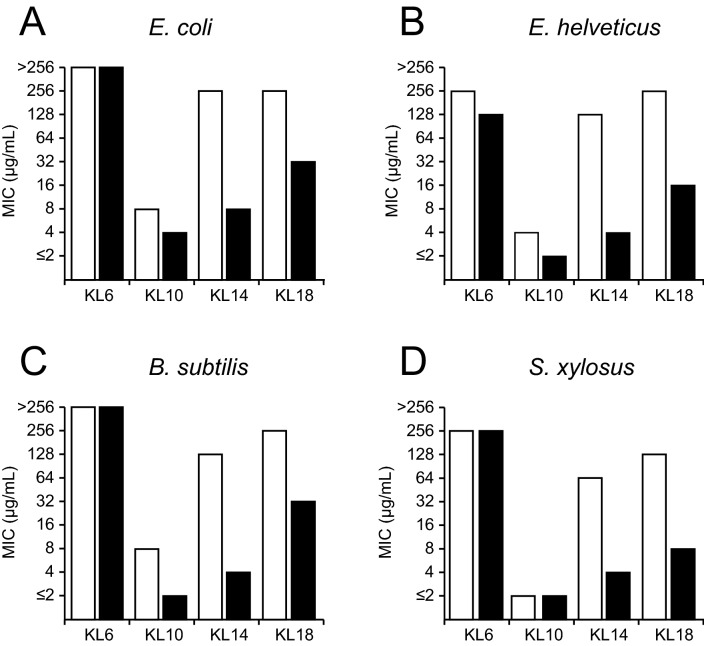
MIC values (µg/mL) for KL peptides in four bacterial strains. Peptides were less active in assay A (white), where they are exposed for a considerable time to the phosphate-containing medium, than in assay B (black) which measured the intrinsic peptide activity. See text for details.

**Figure 6:**
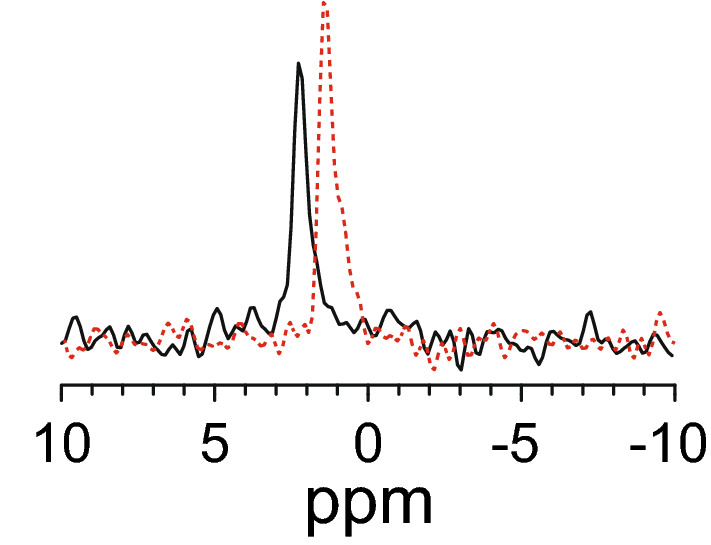
^31^P-NMR spectra of the MH medium used in the MIC tests (black, full line) and of 10 mM phosphate buffer (red, dashed line).

As seen in Fig. 5 (and Table S1), the peptides show an unusual length dependence in their ability to inhibit bacterial growth. In all four tested strains, KL6 is completely inactive, while KL10 is the most active peptide with a MIC of 2–8 μg/mL against the four tested strains. KL14 and KL18 are less active than KL10, but more active than KL6. The peptides show a very similar pattern of activity against the four different types of bacteria, i.e. against Gram-negative (*E. coli, E. helveticus*) and Gram-positive (*B. subtilis, S. xylosus*) strains alike. However, peptide length does not seem to correlate with activity. Interestingly, we found that there are distinctive differences in the actual MIC values, depending on the way the assay is being performed. In the standard assay (assay A, white bars), which our group and many other labs have routinely used in numerous previous studies^11,13,35^, the peptide is first mixed with the growth medium in the wells on a microtiter plate to form a two-fold dilution series, and then the bacteria in medium are added to each well. In this case, we pondered, the peptide might be exposed to phosphate ions in the growth medium and start to misbehave. We therefore conceived a different order of mixing in assay B (black bars), such that the two-fold dilution series is prepared with peptides in MilliQ water (pH ≈ 6), and then bacteria are added to each well in doubly concentrated medium. More details are given in the Methods section. The main difference is that in assay A, peptides are in contact with the growth medium for at least an hour before the bacteria are added, whereas in assay B the peptides get into contact with the bacteria and medium at the same time. Since it was shown above that the KL peptides with at least 10 amino acids aggregate in the presence of phosphate (Fig. 1C), we needed to find out whether there is any phosphate in the commercial growth medium. The exact composition of the Müller-Hinton broth is neither declared nor easily determined, but we estimated the phosphate content of the solution using ^31^P-NMR. Two samples were prepared, one with 10 mM phosphate buffer, and one with Müller-Hinton medium at twice the final concentration (i.e. the stock used in version B of the MIC assay). NMR spectra were recorded of the two samples using identical experimental conditions. From the ^31^P-NMR spectra (Fig. 6) it can be seen that the doubly concentrated medium gives a signal similar to that of the phosphate buffer, indicating a phosphate content of 8–10 mM. Part of this may be in the form of nucleic acids or other compounds, but large molecules would probably give broader lines. As the line width is similar to that of free phosphate, we assume that most of the signal comes from free phosphate ions in solution. Thus, in version A of the growth inhibition assays, the peptides are exposed to a phosphate concentration of 4–5 mM in the standard growth medium for over an hour before bacteria are added.

When the results of assay A and assay B are compared, it is obvious that the longer KL peptides are much more active in assay B than in assay A (Fig. 5). For KL6, there is hardly any change and the peptide is completely inactive in both assays—which is not surprising in view of its general lack of folding. KL10 is more active in assay B by a factor 2–4, with the largest effect in *B. subtilis* (Fig. 5C). KL14 shows a dramatic difference, giving 16–32 times lower MIC values (i.e. higher activity) in assay B. KL18 is also much more active in assay B, with 8–16 times lower MIC values. We may thus interpret assay A to be affected by phosphate-induced aggregation artefacts, leading to an effectively lower concentration of (non-aggregated) peptide that can bind to and permeabilize the bacterial membranes. Assay B, on the other hand, may be regarded as a measure of the genuine antimicrobial activity of the KL peptides. In this context it is interesting to note that KL10 is the most active molecule in assay B, yet the activities of the longer peptides are only moderately lower (around fourfold). In the artefact-ridden assay A, on the other hand, MIC of KL10 is typically 32 times smaller than for KL14, leading to a much more dramatic appearance of the differences.

### Hemolysis

**Figure 7:**
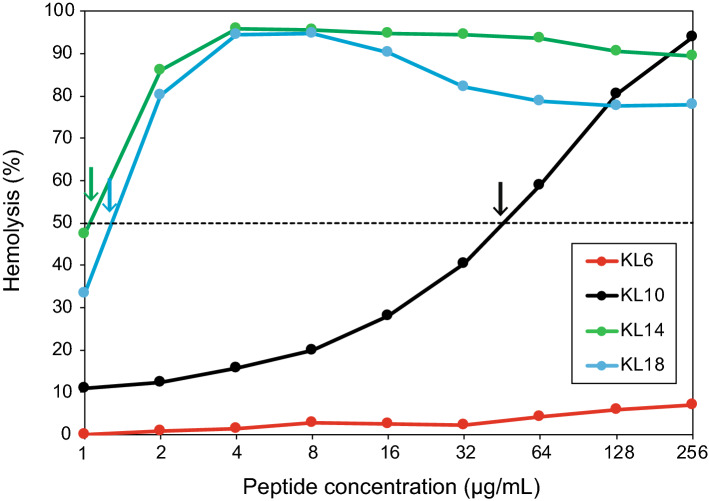
Hemolysis for KL peptides as a function of peptide concentration. The dotted line indicates 50% hemolysis, and the arrows indicate the HC_50_ values.

Amphiphilic antimicrobial peptides usually show membranolytic effects against not only bacteria, but they also tend to permeabilize eukaryotic cells such as erythrocytes. Hemolytic activities of the KL peptides are summarized in Fig. 7 (and in Supplementary Table S2), recorded for several different peptide concentrations. KL6 gives almost no hemolysis, even at the highest tested concentration. KL10 becomes moderately hemolytic with increasing concentration, giving 50% hemolysis at around 50 µg/mL. KL14 and KL18 are very hemolytic and give more than 80% hemolysis already at 2 µg/mL. This gives an order for HC_50_ (the peptide concentration giving 50% hemolysis) of KL6 (> 256 µg/mL) >> KL10 (≈ 50 µg/mL) > KL14 (≈1.3 µg/mL) > KL18 (≈1 µg/mL). For these two longest peptides, hemolysis seems to become reduced again at higher concentrations, presumably because the peptides start to aggregate under those conditions and are no longer available for membrane interactions—even in the absence of added phosphate buffer in this assay. Note that the hemolysis assay is carried out in Tris-HCl buffer at pH 7.6 over 30 min, while standard MIC tests utilize 4–5 mM phosphate at pH 7.3 and take overnight (20 h).

### Vesicle leakage

In the MIC and hemolysis assays above with living cells, it is not possible to define or control the composition of the membrane lipids, and the lipid acyl chain lengths are highly heterogeneous. Therefore, we also performed complementary in vitro experiments by measuring the leakage of fluorescent dye from small unilamellar vesicles with a well-defined lipid composition. The mixture POPC/POPG (1/2 mol/mol) of zwitterionic phosphatidylcholine (PC) and anionic phosphatidylglycerol (PG) head groups was used, because a negative lipid charge is necessary to attract the water-soluble cationic peptides electrostatically to the vesicles, as seen in the CD experiments. Furthermore, anionic lipids are known to be the main components of bacterial membranes, which contain in many cases well over 50% PG^36^. Leakage curves were measured over a period of 10 min after mixing the vesicle suspension with the peptide solution. The experiments were performed under several different conditions in order to systematically investigate the aggregation behavior of peptides observed above.

Peptide stock solutions were always kept in MilliQ water (pH ≈ 6) to avoid aggregation. Vesicles were prepared in a dye-containing buffer (either phosphate or PIPES, pH 7) as multilamellar vesicles (MLVs), and then extruded to give large unilamellar vesicles (LUVs, diameter ~ 120 nm). Immediately before the leakage measurement, they were subjected to gel filtration in order to remove the dye from outside the vesicles. The filtered LUVs were diluted in the corresponding buffer to 100 μM concentration. Upon mixing the peptides and vesicles, fluorescence dequenching was monitored for 600 s to reflect leakage. Finally, detergent was added to micellize the vesicles and give a reference value corresponding to 100% leakage.

**Figure 8:**
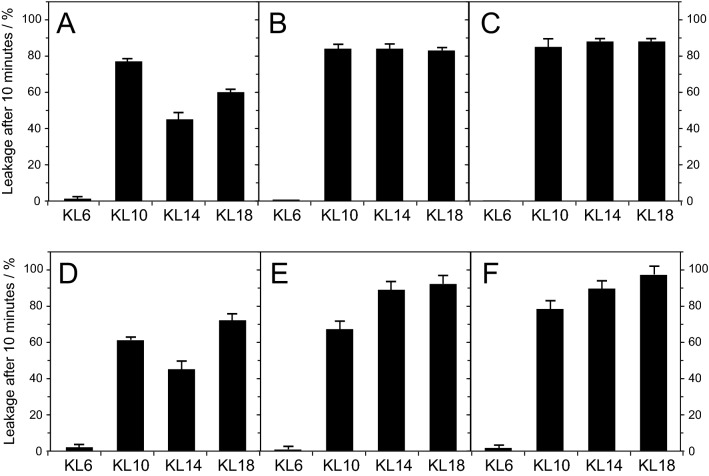
Leakage of POPC/POPG (1/2) vesicles induced by KL peptides. Panels A, B, C, were performed with a constant weight of 30 µg peptide acting on 115 µg lipids in a 1,500 µL volume. Panels D, E, F were performed with a constant molar peptide concentration of 10 µM and 100 µM lipids. (**A**, **D**) Vesicles in phosphate buffer (PB) were added to peptides in PB. (**B**, **E**) Peptides in water were added to vesicles in PB. (**C**, **F**) No PB was used, as the peptides in water were added to vesicles in PIPES buffer.

Leakage experiments were performed with a P/L of 1/10 for KL14 and the same mass ratio for the other peptides (in the 1,500 µL cuvette 30 µg of peptide and 115 µg of lipids were used). In panel A (Fig. 8A), gel filtration was done in phosphate buffer (PB), the peptide was added first to the cuvette containing PB, and after 100 s POPC/POPG (1/2) vesicles in PB were added. In this case, KL6 gave essentially no leakage, KL10 gave the highest leakage, whereas the longer peptides were less active. In panel B (Fig. 8B), gel filtration was done in PB, but now the lipid vesicles were first added to the cuvette containing PB, while the peptide was added after 100 s. In this case, KL6 gave no leakage, KL10 gave over 80% leakage, and the same activity was found for KL14 and KL18. In panel C (Fig. 8C), there were no phosphate ions present at all, as vesicle preparation and gel filtration were done in PIPES buffer, and lipid and peptide were also suspended in this buffer. In this phosphate-free set-up, the same leakage values were found as in panel B, where the peptide was not exposed to PB before having a chance to encounter the vesicles. Experiments were also performed with a constant P/L of 1/10 (Fig. 8D–F), which is commonly used to compare peptides. In these measurements, KL10 was less active and KL18 more active than in the constant mass series. Since the mass ratio in this case was lower for KL10 and higher for KL18, the results are compatible with the result using a constant peptide-to-lipid mass ratio. The results show that in absence of phosphate, all the peptides with 10 or more amino acids gave the same leakage, indicating a very similar intrinsic activity for the same number of [KL] units for all peptides. The unusual behavior of KL6 will be discussed below.

### Binding affinity of peptides to vesicles

Peptide binding to POPC/POPG (1/1) vesicles was measured using a fluorescence assay, which is based on the difference in fluorescence of NBD-labeled peptides when they are bound to the vesicles and when they are free in aqueous solution. Details are given in the Supporting Material, and the results are presented in Table 3. It was found that all peptides bind strongly to the vesicles, with partitioning constants K_p_ > 100,000 in all cases. KL6 has the weakest and KL18 the strongest binding; KL10 was found to have a stronger binding than KL14 (but note that the error bars are considerable).

**Table 3:** Partitioning constants, K_p_, of KL peptides towards POPC/POPG (1/1) vesicles, calculated from the fluorescence binding curves of NBD-labeled peptides.

Peptide	K_p_
KL6	138,000 ± 12,000
KL10	365,000 ± 46,000
KL14	255,000 ± 23,000
KL18	503,000 ± 67,000

Average values and standard deviations are given.

## Discussion

Antimicrobial peptides are often cationic and tend to have the ability to fold into amphipathic structures. These features enable them to bind avidly to negatively charged membranes of microorganisms as amphipathic α-helices or β-sheets, which can permeabilize the membrane and kill the cells. Much attention has been devoted to synthetic model peptides with these properties (c.f. Introduction section), especially sequences with a regular pattern of cationic and hydrophobic residues. Here, we have prepared a series of KL peptides with different lengths, in order to systematically investigate the length dependent membranolytic activity of amphipathic β-strands. Similar peptides have been studied before in the literature, but usually either the peptides were studied in solution without membranes, or only the biological activity was examined, whereas we here combine biological assays (MIC, hemolysis) with biophysical studies in lipid vesicles (CD, leakage) and TEM to get a deeper understanding of their activity and mechanism of action. It is important to note that we can compare the activities of all our peptides based on the same mass, rather than using a constant molar concentration. Therefore, any length-dependence observed here will reflect the intrinsic activity of the particular peptide molecule, and cannot be simply attributed to the increasing molecular weight or increasing charge of a peptide with increasing length.

The antimicrobial activity of KL-like peptides has already been explored in the literature, though not of the exact sequences used here. In an early study, KL18 (but acetylated at the N-terminus and amidated at the C-terminus, hence with less charge than our amidated KL18), was found to be inactive against bacteria^9^. Later, KL9, KL11 and KL15 (sequences [KL]_4_K, [KL]_5_K, and [KL]_7_K, all dansylated at the N-terminus) were studied, and KL9 turned out to be the most active, while KL11 and KL15 had lower but similar activities (based on constant molar concentration)^17^. Hemolysis of these three peptides, on the other hand, was reported to increase with peptide length (based on constant molar concentration)^37^. These peculiar observations fit with our results, and we can now explain why KL-type peptides with about 10 amino acids show the highest antimicrobial activity in standard MIC assays.

Using CD to monitor the generation of secondary structure from a random coil state in MilliQ water (pH ≈ 6), we found that KL6 is too short to form β-sheet structures under any conditions tested here. KL10 and longer peptides are also unstructured in MilliQ water, but they form β-structures when the pH of the solution is increased to around 10, or in the presence of phosphate ions (10 mM PB, pH = 7), or in the presence of anionic phospholipid vesicles (Fig. 1). Time-resolved CD analysis demonstrated that the longer KL peptides aggregate strongly in the presence of PB, and the speed of aggregation increases with peptide length and phosphate concentration (Fig. 2). On the other hand, peptides do not aggregate in Tris buffer, nor in the presence of NaF or K_2_SO_4_ salts without buffer. This indicates that there must be some specific interaction with the polyvalent phosphate anions that leads to aggregation of the polycationic KL peptides. In the present study, relatively low salt concentrations (around 10 mM) were used, although previously it had been observed that KL peptides can form β-structures under high NaCl concentrations of 100 mM or more^19,21^.

KL14 and KL18 aggregate almost completely to β-sheet structures in less than a minute in the presence of 5 mM phosphate. This is the concentration at which the MIC assays is performed, and in the standard procedure the peptides are actually in contact with the phosphate containing medium for over an hour before the bacteria are added. During this time, peptides will certainly aggregate and precipitate, hence it is not surprising that KL14 and KL18 do not show much activity against bacteria under these conditions. Only KL10, which aggregates more slowly, will still be present in solution to a fair extent and able to attack the bacteria. When the MIC assay is modified such that peptides are minimally exposed to phosphate ions before they encounter the bacteria, a much higher activity is found for KL14 and KL18 (Fig. 5). We can thus conclude that these peptides are able to attack the bacteria before they get sequestered by phosphate into inactive aggregates. KL10 is also more active in the modified MIC assay, so even this peptide must be getting partially deactivated by aggregation in the standard protocol.

We predict that the longer peptides would be even more active, if the antimicrobial tests could be done completely without phosphate. However, phosphate-free conditions cannot be tested, because the bacteria need phosphate for growth. As an alternative experiment, we therefore used vesicle leakage experiments in vitro as a simplified version of the MIC assay, testing only the permeabilization of the lipid bilayer. In these fluorescence leakage experiments, a similar response is seen in the presence of PB (Fig. 8A) as in the standard MIC assay (version A, which our group has used in many previous studies): KL6 is completely inactive, KL10 is most active, and the longer peptides show lower activity. However, when the leakage experiment is performed in the absence of phosphate ions, all peptides (except for KL6, as explained below) have a substantial and almost identical activity (Fig. 8C). The same result is obtained if peptides are exposed to vesicles and phosphate simultaneously (Fig. 8B), indicating that they can quickly bind to vesicles and induce leakage before they become aggregated due to phosphate. It can be noted that the leakage experiments are done in buffer containing 150 mM NaCl, which is similar to the conditions used in our biological assays. These results demonstrate that the longer peptides are intrinsically just as active against bacteria as the most promising candidate KL10, but in the conventional MIC assay the longer ones become sequestered due to their avid interaction with phosphate. In fact, in the hemolysis assay, where the peptides are not in contact with phosphate initially, the longer ones show an intrinsically higher activity than the shorter ones (Fig. 7). In the hemolysis assay, the red blood cells are suspended in a phosphate-free solution, but when they start to leak, cell contents including phosphate will escape, so peptides may start to aggregate eventually. This is indeed in line with our observation that the activity of the longer peptides decreases again at higher concentration, most likely due to aggregation.

With the exception of KL6, all other peptides are seen by CD to fold into β-sheeted structures as a function of time, when incubated in (1) MilliQ water at pH 10, (2) in 10 mM phosphate buffer (pH 7), or (3) in the presence of anionic lipid vesicles (DMPC/DMPG 4/1, and POPC/POPG 1/1). Peptide aggregation induced by free phosphate ions thus constitutes the critical process that prevents our peptides from becoming active at the lipid membrane. KL10, KL14 and KL18 are all seen by TEM to form amyloid-like fibrils and ribbons, when incubated for 24 h in MilliQ water at pH 10, or after only short incubation in 5 mM phosphate buffer (Fig. 4). Such self-assembly obviously takes place in the CD and MIC experiments in the presence of PB, and we can conclude that these fibrillar aggregates are inactive and do not permeabilize membranes. Literature data of similar peptides confirm the results reported here^9,17,37^. It is likely that also the KL-type peptides used in those studies, and other similar peptides, aggregate in the presence of phosphate ions, but this was not discussed previously. It is thus crucial for the interpretation of experimental data to be aware of the specific effects of phosphate ions on these kinds of peptides and take them into account in the study design and data analysis.

Our CD analysis suggests that the β-sheet structures formed by the KL peptides in membranes are not identical to but may have a similar local fold as in the amyloid-like fibrils precipitated from aqueous solution. This is consistent with our earlier observation of another amphiphilic model peptide [KIGAKI]_3_^14,16^. Membrane binding is generally initiated by long-range electrostatic attraction and consolidated by hydrophobic interactions of the Leu side chains. In analogy to the KIGAKI system, the KL peptides will also bind initially as monomers, but will become immobilized presumably by lateral self-assembly within the membrane above a certain concentration threshold. The actual membrane perturbation (in antimicrobial tests, hemolysis, and vesicle leakage) must therefore be attributed either to the insertion of the [KL]_n_ strands into the outer monolayer, and/or to further aggregation and immobilization of the membrane-bound peptides. To disentangle these steps from one another, we can learn a lot from the behavior of KL6. This peptide is always inactive, in every assay and under essentially all conditions tested, but according to our binding assay it nevertheless has a high affinity for anionic vesicles (Table 3). We can thus conclude that KL6 is not inactive because it does not bind to membranes. Instead, it is inactive because it is too short to self-assemble in the membrane, while as a monomer it is not disturbing the membrane enough to show activity.

Interestingly, membrane binding of KL-peptides may not be as closely related to vesicle leakage as it might have been expected. It had been previously shown for other types of cationic peptides that even those with very low membrane affinity were still able to induce considerable leakage^38^. Here we find, on the other hand, that KL6 binds well but induces no leakage. Furthermore, it has been observed that anionic membranes can accelerate the aggregation of amyloid peptides^39–41^. Here, we see that KL peptides with at least 10 amino acids form β-sheets in the presence of membranes and also form fibrils in solution. Thus, these KL peptides can be assumed not to be monomeric in the membrane but rather to self-assemble into some kind of relatively immobile β-sheet aggregate. KL6, on the other hand, does not form β-sheets in the membrane, and thus seem to bind as a flexible monomer. This interpretation implies that peptide-aggregation is necessary for membrane activity (MIC, HC50, leakage). Given the high affinity of KL-peptides for phosphate groups, this may actually involve the phospholipids in the bilayer (besides the obvious hydrophobic interactions), and must certainly not occur beforehand in solution.

## Conclusions

**Figure 9:**
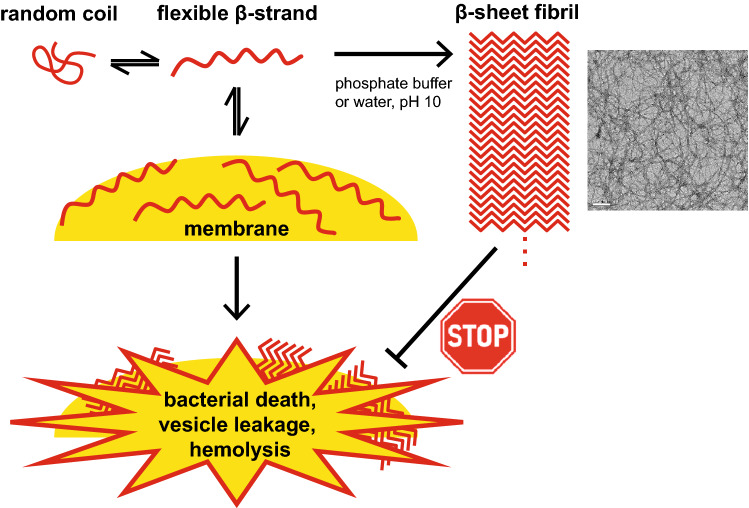
Overview of the pathways taken by KL peptides. The peptides are largely unstructured in water, but they will bind to an anionic lipid bilayer as β-strands that self-assemble further (possibly into bilayer-embedded β-sheets), thereby permeabilizing the membrane. At a sufficiently high concentration, peptides induce leakage of vesicles and kill cells in this manner. However, the presence of phosphate ions or high pH leads to rapid aggregation into free amyloid-like cross-β-sheet fibrils, which are no longer membrane-active.

KL peptides with lengths between 6 and 18 amino acids have been investigated in solution as well as in the presence of model membranes and cells. KL6 binds to anionic lipid bilayers but is too short to form β-sheets and therefore has essentially no membranolytic activity. The results for KL peptides with 10–18 amino acids are summarized in Fig. 9 and in Table 4. In MilliQ water and common salt solutions, the peptides are unstructured, but in the presence of phosphate ions (or at high pH) they tend to self-assemble into amyloid-like β-sheet fibrils and ribbons. The speed of aggregation increases with peptide length and phosphate concentration. The resulting fibrils are no longer available to interact with membranes, thereby reducing the observed activity of that sample. The peptides also have a high affinity for lipids, and self-assembly within the bilayer will permeabilize vesicles and erythrocytes, and kill bacteria. In many assays, two competitive and opposing effects are found to operate in a subtle balance, leading to a peculiar order in the length dependent activity: phosphate-induced aggregation inactivates the peptides, while membrane binding and self-assembly in the bilayer leads to permeabilization. In the vesicle leakage assay without phosphate, peptides of length 10, 14 and 18 amino acids show the same activity, when a constant peptide mass is used, indicating a similar intrinsic activity of the peptides. In conventional antibiotic assays, KL10 shows the highest activity against bacteria, because it is on the one hand long enough to form extended β-sheets on a membrane, while on the other hand it is still short enough to not immediately be driven into inactive fibrils by phosphate ions in solution. With this explanation, the seemingly erratic behavior of the series of KL peptides has been attributed to simple physicochemical and structural factors. Now, it will be possible to better control their activities in different environments, and possibly to design further improved analogues for designated biotechnological or pharmacological applications.

**Table 4:** Summary of aggregation propensity and activities of KL peptides of length 6–18 amino acids, measured at constant peptide mass.

Aggregation in PB (pH 7) or water (pH 10)	**KL6 << KL10 < KL14 < KL18**
Aggregation in presence of lipids	**KL6 << KL10 < KL14 < KL18**
Antimicrobial activity (with phosphate)	KL6 << KL10 >> KL14 ≈ KL18
Antimicrobial activity (reduced phosphate exposure)	KL6 << KL10 > KL14 > KL18
Hemolysis (no initial phosphate)	**KL6 << KL10 < KL14 < KL18**
Vesicle leakage (with PB)	KL6 << KL10 > KL14 < KL18
Vesicle leakage (no phosphate)	**KL6 << KL10** ≈ **KL14** ≈ **KL18**

In bold print are shown the straightforward correlations between length and activity, once phosphate-induced aggregation artefacts have been eliminated.

## Supplementary information

Supplementary information
