# Supplementary material for: Phosphate-dependent aggregation of [KL]n peptides affects their membranolytic activity
Source: Sci Rep. 2020 Jul 23;10:12300. doi: 10.1038/s41598-020-69162-0 (PMC7378186; doi:10.1038/s41598-020-69162-0)
Supplement: Supplementary file 1 — Supplementary information [file 41598_2020_69162_MOESM1_ESM.pdf]

Phosphate-dependent aggregation of $[KL]_n$ peptides affects their membranolytic activity

Erik Strandberg, Fabian Schweigardt, Parvesh Wadhwani, Jochen Bürck, Johannes Reichert, Haroldo L. P. Cravo, Luisa Burger, and Anne S. Ulrich

Supporting information

Supporting Tables

Table S1. MIC values (µg/mL) for KL peptides in four bacterial strains. The highest tested concentration was 256 µg/mL and the lowest 2 µg/mL.

**Peptide**	**Gram negative**		**Gram positive**	
	E. coli	*E. helveticus*	*B. subtilis*	*S. xylosus*
**Assay A**				
KL6	> 256	256	> 256	256
KL10	8	4	8	≤ 2
KL14	256	128	128	64
KL18	256	256	256	128
**Assay B**				
KL6	> 256	128	> 256	256
KL10	4	≤ 2	≤ 2	≤ 2
KL14	8	4	4	4
KL18	32	16	32	8

Table S2. Hemolysis for KL peptides. Values are in % relative to total hemolysis (100%) after addition of triton-X100.

**Peptide concentration (µg/mL)**	**Peptide**			
	**KL6**	**KL10**	**KL14**	**KL18**
1	0	11	47	33
2	1	12	86	80
4	1	16	96	95
8	3	20	96	95
16	2	28	95	90
32	2	40	94	82
64	4	59	94	79
128	6	81	91	78
256	7	94	89	78

Supporting Figure

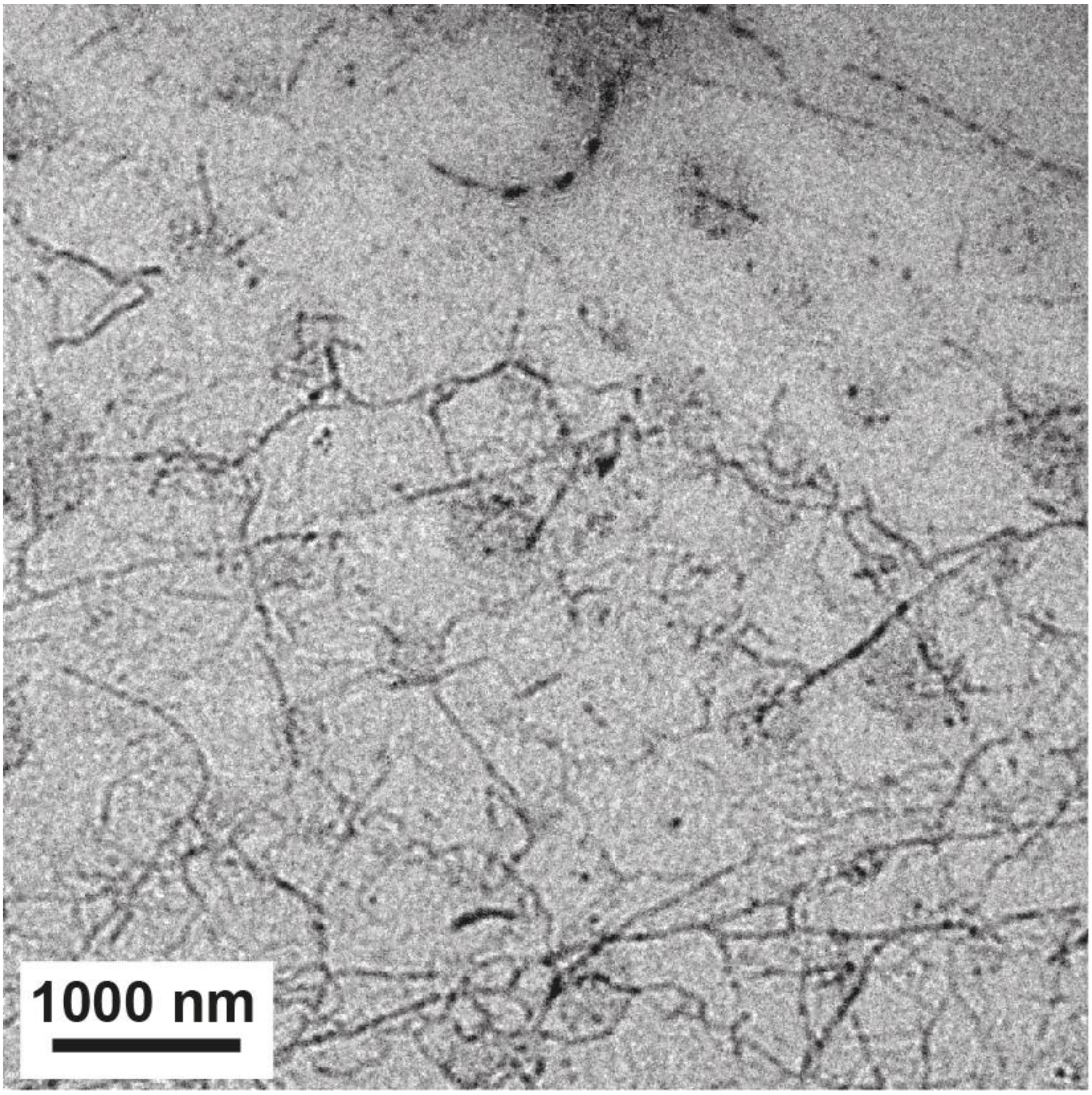

Figure S1. TEM picture of KL10 in water at pH ≈ 10 after 24 h, followed by sonication for 2 min in a low-power sonication bath. Short fibril fragments are observed, indicating that the fibrils seen in **Figure 4** are not very stable.

Description of vesicle binding assay

Vesicles were prepared as described in the methods section for the leakage assay. However, the same buffer was used inside and outside the vesicles, and gel filtration was not performed. The buffer contained 150 mM NaCl and 10 mM HEPES at pH 7.5. Fluorescence measurements were performed in a thermostated cuvette with constant stirring at 30ºC on a FluoroLog3 spectrofluorimeter (HORIBA Jobin Yvon, Unterhaching, Germany). The excitation wavelength was 464 nm and a spectrum was recorded from 480 to 700 nm. At first only buffer was measured as a blank. Then peptide in buffer was measured to get a spectrum of free peptide. Thereafter a series of measurements were performed with a constant peptide concentration of 0.1 µM and varying lipid concentrations. Typically, the first measurement was done with 500 µM lipids to get a spectrum of fully bound peptides, and then additional samples were used with lower lipid concentration. For each sample first the vesicle spectrum was measured, which was subtracted from the spectrum with added peptide. A waiting time of 3 minutes was used between addition of vesicles and measurement, and between addition of peptides and measurements, to give the system time to equilibrate. Experiments were repeated three times and the individual values of K_p were averaged.

Binding data analysis

The binding determination is based on the difference in fluorescence intensity F for the NBD fluorophore when the peptide is dissolved in water (F_W) or bound to the lipid membrane (F_L). The molar fraction of lipid-bound peptide X_L at a given lipid concentration C_L is given by

$$X_L = (F(C_L)-F_W)/(F_L-F_W) \qquad \text{(S1)}$$

According to Ref. [1], X_L can also be obtained as

$$X_L = C_L K_p \gamma_L/(1 + C_L K_p \gamma_L) \qquad \text{(S2)}$$

where K_p is the partitioning constant and γ_L is the molar volume of the lipids in the liquid crystalline phase [1]. As a good approximation the density of the liquid crystalline phase is similar to that of water [2], meaning that with a lipid molecular weight of 760 g/mol for POPC and 771 g/mol for POPG, we get $\gamma_L = 0.765\ M^{-1}$. Combining Eqs. S1 and S2 and rearranging, we get [3]:

$$F(C_L) = (F_W + F_L C_L K_p \gamma_L)/(1 + C_L K_p \gamma_L) \qquad \text{(S3)}$$

By subtracting from both sides the fluorescence of peptides in water without lipids present, we

get

$$F(C_L) - F_W = (F_W + F_L C_L K_p \gamma_L)/(1 + C_L K_p \gamma_L) - F_W(1 + C_L K_p \gamma_L)/(1 + C_L K_p \gamma_L) =$$
$$(F_W + F_L C_L K_p \gamma_L - F_W - F_W C_L K_p \gamma_L)/(1 + C_L K_p \gamma_L) =$$
$$(F_L - F_W) C_L K_p \gamma_L /(1 + C_L K_p \gamma_L) \quad \text{(S4)}$$

A fit of experimental data at different lipid concentration C_L was made using Eq. S4 and from the best fit the partitioning constant K_p was determined. It is possible to calculate the association (or binding) constant K_a, which is related to K_p according to [1]:

$$K_a = \gamma_L K_p \quad \text{(S5)}$$

The fit was performed using a method to find the curve closest to the data points in two dimensions. The data was normalized to (F_L-F_W) along the y axis and the maximum used lipid concentration along the x axis, and the distance from each data point to the closest point on the curve was calculated from a least-squares fit. The sum of these distances was minimized by a grid-search of the fitting parameters F_L and K_p.

Supporting References

1. Melo, M.N. et al. Prediction of antibacterial activity from physicochemical properties of antimicrobial peptides. *PLoS One* **6**, e28549 (2011).
2. Nagle, J.F. & Tristram-Nagle, S. Structure of lipid bilayers. *Biochim. Biophys. Acta* **1469**, 159-95 (2000).
3. Santos, N.C., Prieto, M. & Castanho, M.A.R.B. Quantifying molecular partition into model systems of biomembranes: an emphasis on optical spectroscopic methods. *Biochim. Biophys. Acta* **1612**, 123-35 (2003).
